# Cholinium-Based Ionic Liquids Modulate Protein Stability: A Comparative Study of Enzymes and Albumins

**DOI:** 10.3390/molecules30071574

**Published:** 2025-03-31

**Authors:** Artashes A. Khachatrian, Timur A. Mukhametzyanov, Ramazan Z. Salikhov, Alexandra E. Klimova, Dmitry G. Yakhvarov, Bulat F. Garifullin, Olga S. Terenteva, Pavel L. Padnya, Ivan I. Stoikov, Boris N. Solomonov

**Affiliations:** 1 Department of Physical Chemistry, Kazan Federal University, Kremlyovskaya 18, Kazan 420008, Russia; timur.mukhametzyanov@kpfu.ru (T.A.M.); alexandra_klmv@mail.ru (A.E.K.); yakhvar@iopc.ru (D.G.Y.);; 2 Arbuzov Institute of Organic and Physical Chemistry, FRC Kazan Scientific Center of RAS, Arbuzov St. 8, Kazan 420008, Russia; 3 Department of Organic and Medicinal Chemistry, Kazan Federal University, Kremlyovskaya 18, Kazan 420008, Russiaivan.stoikov@mail.ru (I.I.S.)

**Keywords:** ionic liquid, binding, structure, enzyme, protein

## Abstract

This work aims to assess the intermolecular interaction of choline ionic liquids (ILs) (choline malonate ([Ch][Mal]), choline succinate ([Ch][Suc]), and choline valinate ([Ch][Val]) with two enzymes (lysozyme and α-chymotrypsin). We evaluated the state of the tertiary protein structure using circular dichroism (CD) spectrometry and quantified the binding parameters of the binding of the ionic liquids to the enzymes by fluorescence spectroscopy. The binding energies of the enzymes and the localization of ions on them were estimated using the molecular docking. We then analyzed the relationship between the enzymes’ thermostability and their tendency towards aggregation in the enzyme/ionic liquid systems. The obtained results were compared with previous data on albumins to identify similarities and differences between the behavior of enzymes and albumins in ionic liquid solutions. Despite the comparable values of the binding constants, the effect of ionic liquids on the thermostability of enzymes was the opposite of their effect on albumins. In addition, although these ionic liquids promoted aggregation in both enzymes and albumins, this effect was much more pronounced for albumins.

## 1. Introduction

Ionic liquids have attracted the attention of scientists for several decades due to their unique physical and chemical properties and their wide range of applications [1,2,3,4]. Of particular interest are bio-based ionic liquids, which are highly biocompatible and biodegradable, making them promising compounds for use in biomedical and pharmaceutical applications [5,6]. Bio-based ionic liquids can interact with biomolecules in various ways and can be used to enhance the long-term storage and transportation of DNA, RNA, proteins, enzymes, etc. [7,8]. The study of the thermodynamic parameters for the interactions between ionic liquids and biomolecules is necessary to optimize their structure and composition for specific applications [9,10,11].

Recently, there has been a growing number of studies focused on the thermodynamic properties of biomolecules in aqueous solutions of ionic liquids [12,13,14,15]. Such systems combine environmental safety and functionality, which aligns with the principles of green chemistry [16,17,18,19]. A toxicity study of amino acid-based ionic liquids showed that the size of an anion influences its toxicity—larger anions are more toxic, whereas smaller anions exhibit an improved biocompatibility and biodegradability, and choline-based ILs were found to be virtually harmless to various bacteria [20]. Using thermal and spectroscopic methods, it was confirmed that [Ch][Trp] better promotes the formation of a compact protein structure than [TEA][Trp], especially at higher concentrations. Molecular modeling has demonstrated that [Ch][Trp] forms more hydrogen bonds with proteins (BSA and HSA), which enhances their thermal stability [21]. A study of IL/BSA interactions showed that anions play a key role in protein stabilization; the protein/IL binding strongly depends on the hydrophilicity of the anion. In [22], the binding properties of choline- and amino acid-based ionic liquids and their mechanisms of interaction with DNA were investigated. It was shown that ILs stabilize the DNA structure and have a low binding energy (4 kcal/mol). The DNA/IL interaction was found to occur in the minor groove of DNA. Additionally, choline and amino acid-based ionic liquids have been shown to preserve the structural integrity of RNA and to serve as non-toxic, biocompatible solvents for RNA [23].

In [24], it was observed that, at low concentrations, choline and amino acid-based ionic liquids ([Ch][Pro] and [Pro][NO_3_]) preserve the secondary and tertiary structures of cytochrome c without affecting its thermal stability. These interactions involve both electrostatic and hydrophobic forces, with [Pro][NO_3_] having a weaker effect on the protein than [Ch][Pro]. A comparison of [Ch][Gly] and [Ch][Br] revealed that amino acid-based ionic liquids such as [Ch][Gly], where both ions are of biological origin, have a stronger negative effect on the structure and activity of enzymes than [Ch][Br]. These studies highlight the differences between ILs in their capacity to stabilize proteins and other biomolecules. Consequently, selecting the optimal IL for a specific task is critical for biomedical applications [25].

The strategic selection of anions can further enhance the “green” and biocompatible characteristics of the resulting ionic liquids, particularly given that choline itself is non-toxic. In this context, anions of amino acids (e.g., valine) and organic acids (e.g., malic and succinic acids) are promising. Valine, as an amino acid, contains a proton donor functional group, while malic acid and succinic acid each possess two carboxyl groups, allowing for the formation of cyclic hydrogen-bonded complexes. These factors may potentially improve the solubility of the resulting ILs in water and could potentially stabilize enzymes via hydrogen bonding; however, given that the proteins and enzymes in these formulations have substrate specificity, there is a need to experimentally test the effects of novel ILs.

**Figure 1:**
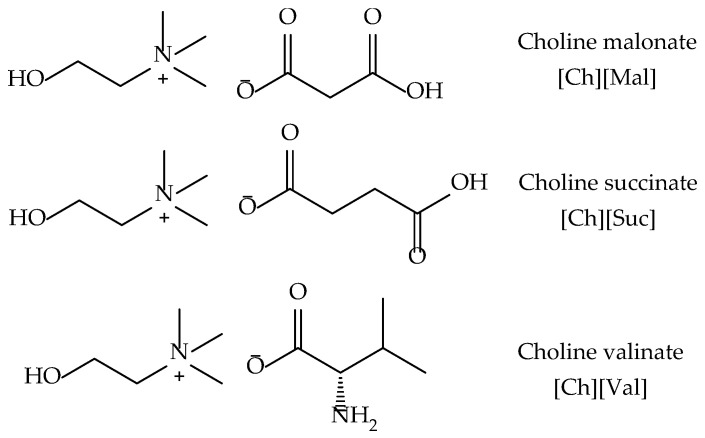
The structures of the choline-based ionic liquids that were studied.

In this work, we investigated the interaction of choline malonate [Ch][Mal], choline succinate [Ch][Suc], and choline valinate [Ch][Val] with two enzymes (lysozyme and α-chymotrypsin). The structures of the studied ILs are presented in Figure 1.

In the current work, the thermal stability of these enzymes in the presence of ILs was examined by analyzing the temperature dependence of their circular dichroism spectra. The binding constants of these enzymes in aqueous IL solution were determined using fluorescence spectroscopy. Additionally, the binding energies of the IL ions bound to the enzymes were estimated using the molecular docking method. Dynamic light scattering (DLS) was employed to measure the hydrodynamic diameters of the aggregates formed by the enzymes and ILs. The interaction of these ILs with serum albumin (human and bovine) has been previously studied [26]. Here, we comparatively analyzed the thermal stability, binding, and aggregation of protein molecules of different natures (enzymes and albumin). Elucidating the patterns of these proteins in binding with these ILs is crucial for developing novel methodologies of protein isolation and stabilization, enabling the development of sustainable enzymatic reaction systems and advancing other applications of ILs in pharmaceutics and biotechnology.

## 2. Results

### 2.1. Circular Dichroism Spectroscopy

**Figure 2:**
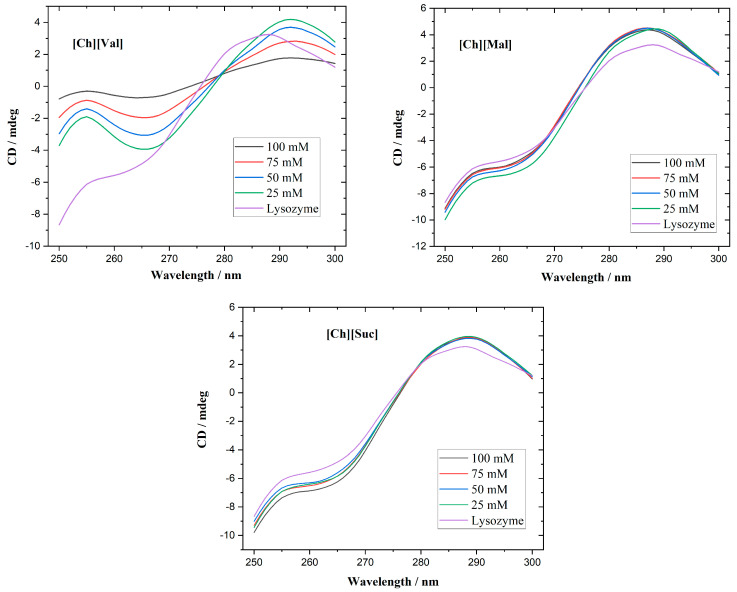
CD spectra of lysozyme with ionic liquids at different concentrations.

Circular dichroism spectroscopy is commonly employed to track alterations in protein secondary and tertiary structures. While the far-UV range (200–250 nm) is optimal for studying secondary structures, the strong absorption of the investigated ionic liquids (ILs) in that region complicates such measurements. Consequently, we focused on the near-UV CD region (250–300 nm), which is sensitive to the microenvironment of aromatic residues and thus reflects the tertiary structure. Native lysozyme typically exhibits a positive triplet in the 280–300 nm region [27], originating from tryptophan, tyrosine, and disulfide bridge-contributions [28]. As shown in Figure 2, its near-UV CD-spectra displays three positive bands at 283, 290, and 294 nm. At 25 mM, for the [Ch][Val] solvent, a partial loss of the tertiary structure of the enzyme is evident. Further increasing the concentration intensifies the unfolding, finally resulting in nearly complete tertiary structure disruption at 100 mM. In contrast, adding [Ch][Suc] and [Ch][Mal] produces negligible changes to lysozyme’s near-UV CD spectra, suggesting that the native conformation remains largely intact.

**Figure 3:**
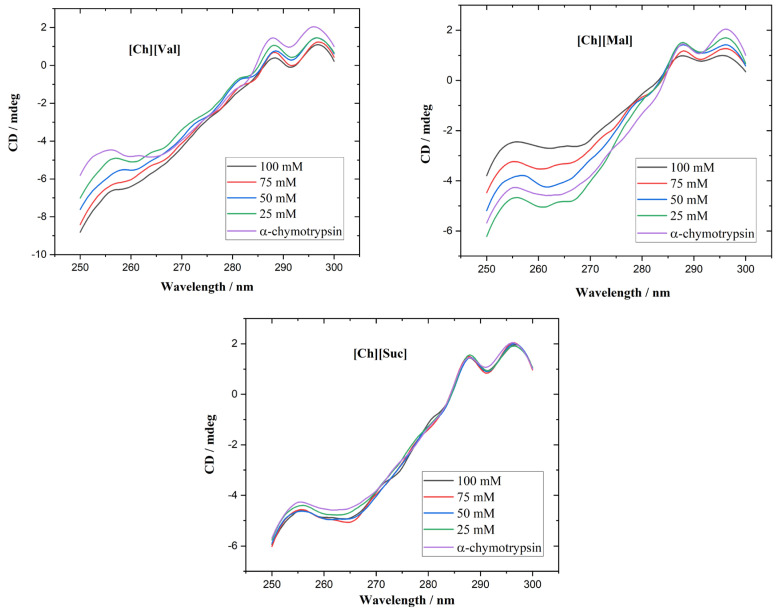
CD spectra of a-chymotrypsin with ionic liquids at different concentrations.

Figure 3 presents the near-UV CD spectra of a-chymotrypsin, which include two positive maxima at 288 and 297 nm and two negative maxima at 291 and 305 nm [29]. Upon raising the concentrations of [Ch][Val] and [Ch][Mal], changes in α-chymotrypsin’s tertiary structure become apparent. Higher concentrations of [Ch][Val] cause both the positive and negative peaks to shift toward more negative values, hinting at partial tertiary structure disruption. Similarly, increased [Ch][Mal] concentrations diminish the intensities of these peaks, signifying unfolding and reduced microenvironmental asymmetry. However, high concentrations of [Ch][Suc] do not alter the enzyme’s near-UV CD spectra, indicating the retention of its native tertiary structure.

### 2.2. Temperature Circular Dichroism Scans of Enzymes in the Solutions with Ionic Liquids

**Figure 4:**
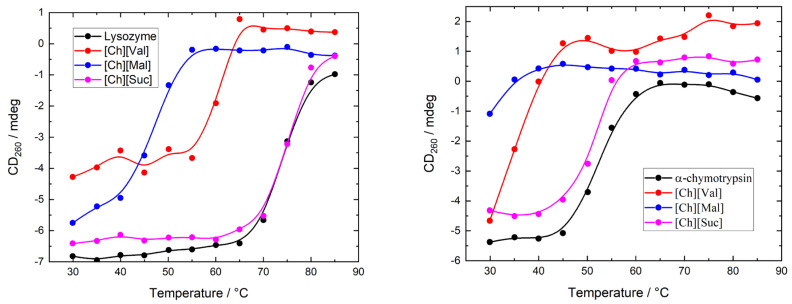
The CD signal at 260 nm as a function of temperature for enzyme/IL system.

**Figure A1:**
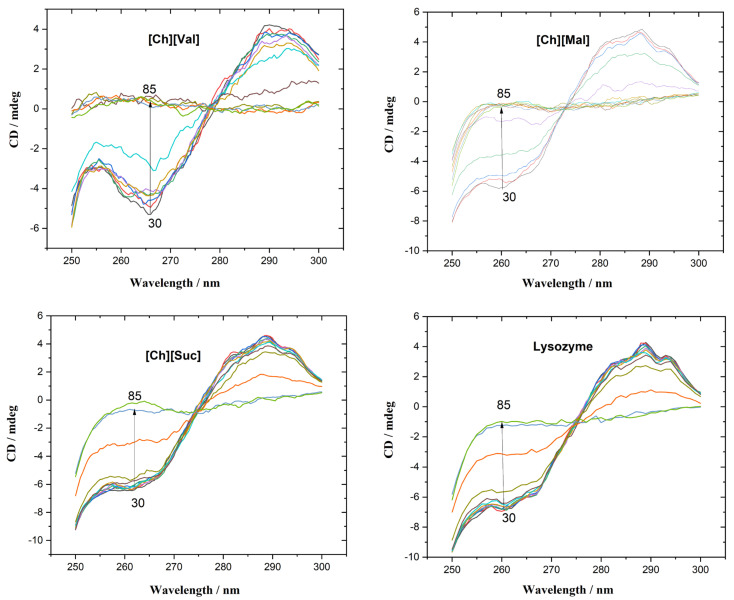
The CD spectra of lysosyme with ionic liquids at different temperatures, Black—30°, red—35°, blue—40°, green—45°, purple—50°, brown—55°, light blue—60°, dark brown—65°, dark yellow—70°, light red—75°, light blue—80°, light green—85°.

**Figure A2:**
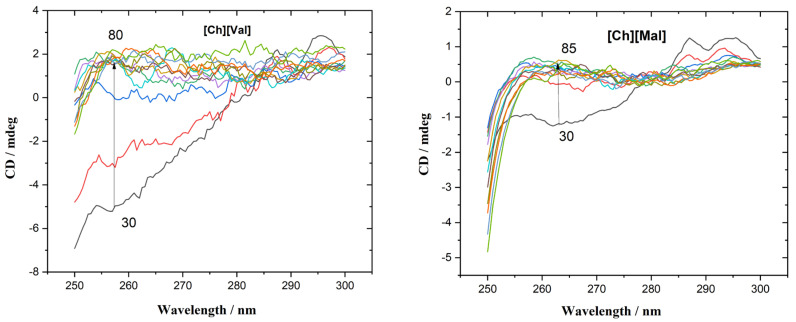

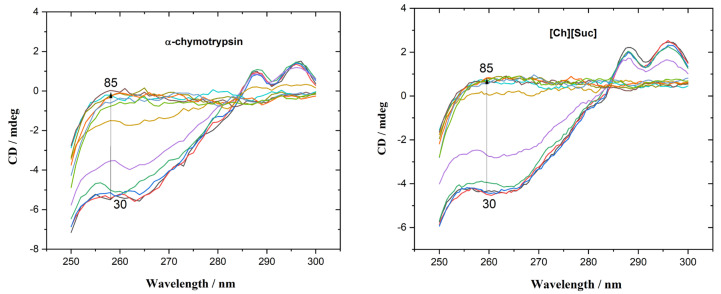
The CD spectra of a-chymotrypsim with ionic liquids at different temperatures, Black—30°, red—35°, blue—40°, green—45°, purple—50°, brown—55°, light blue—60°, dark brown—65°, dark yellow—70°, light red—75°, light blue—80°, light green—85°.

To evaluate the influence of ionic liquids on the stability of enzymes, we measured the temperature-dependent circular dichroism (CD) spectra of lysozyme and α-chymotrypsin heated from 30 °C to 85 °C in 5 °C increments. The concentration of ionic liquids was 0.1 M in all experiments. The resulting CD spectra are shown in Figure A1 and Figure A2. Figure 4 illustrates the temperature-induced variations in the CD signal of the enzyme/ionic liquid mixtures at 260 nm, where it can be seen that the variations in ellipticity correspond to the changes in the enzyme’s tertiary structure. Adding [Ch][Suc] had a negligible impact on the thermal unfolding profiles of both enzymes. In contrast, [Ch][Val] and [Ch][Mal] shifted the onset of unfolding to lower temperatures as compared to the enzyme-only solutions (5 mg/mL of enzyme dissolved in 10 mM of PBS buffer), indicating that these ionic liquids destabilize the native conformations of lysozyme and α-chymotrypsin.

### 2.3. The Binding Between Enzymes and Ionic Liquids

**Figure 5:**
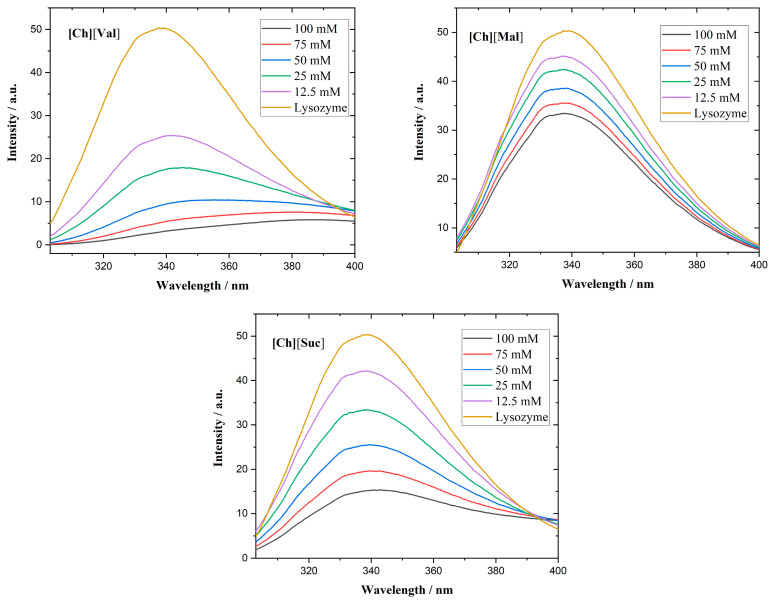
Fluorescence emission spectra of lysozyme in the presence of different concentrations of ionic liquids.

We employed fluorescence spectroscopy to determine how ionic liquids (ILs) associate with various enzymes. Figure 5 shows the fluorescence emission spectra of lysozyme in the presence of ILs. Although lysozyme possesses six Trp residues, only two determine the majority of its fluorescence, as they lie near the enzyme’s active center [30].

In all investigated lysozyme/IL systems, quenching of the fluorescence emission of lysozyme is observed. Specifically, [Ch][Val] induces a red shift of the emission maximum, suggesting structural unfolding and an increase in the polarity of the microenvironment near the Trp residues [31]. Supporting the circular dichroism results, fluorescence measurements revealed that adding [Ch][Val] to lysozyme solutions unfolds the enzyme’s tertiary structure. In contrast, [Ch][Suc] and [Ch][Mal] do not cause the shifts in the emission wavelength.

**Figure 6:**
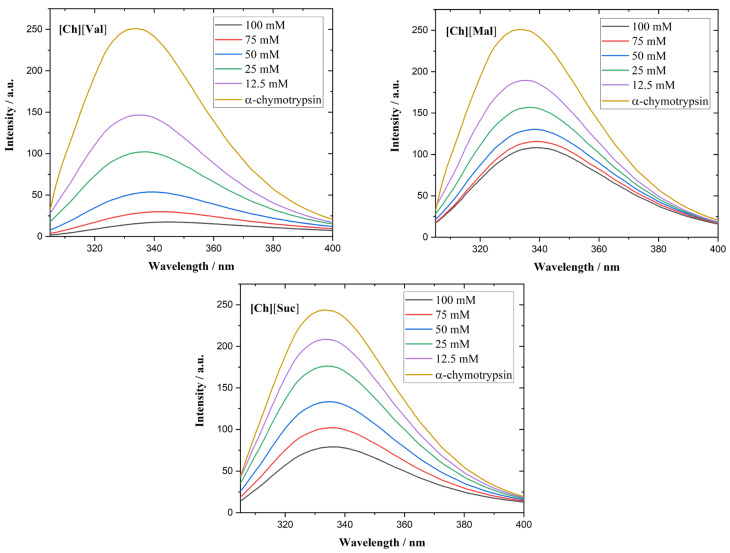
Fluorescence emission spectra of a-chymotrypsin in the presence of different concentrations of ionic liquids.

The α-chymotrypsin spectra of the ILs appear in Figure 6. This enzyme contains eight Trp residues and displays a fluorescence maximum at 333 nm [32]. In the presence of [Ch][Val] or [Ch][Mal], a red shift in the emission maximum emerges, corroborating the circular dichroism results that show that these ILs disrupt the enzyme’s tertiary conformation.

The fluorescence quenching in the enzyme/IL systems was calculated using the Stern–Volmer relationship, shown in Equation (1).

F_0_/F = 1 + K_SV_[D] (1)

log((F_0_ − F)/F) = log(K_b_) + n log[D] (2)

**Figure 7:**
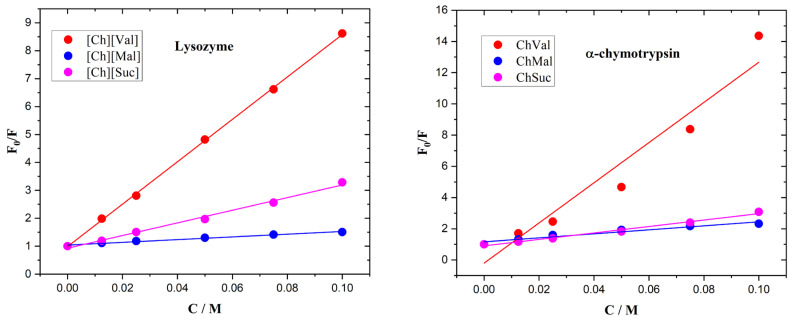
Stern–Volmer plots of lysozyme and a-chymotrypsin with ionic liquids.

In the equations above, F_0_ and F are the fluorescence intensities of the solution with and without IL, D is the IL concentration, Ksv is the quenching (Stern–Volmer) constant, Kb is the binding constant, and n is the number of binding sites. Stern–Volmer plots of the enzymes with ionic liquids are presented in Figure 7. The calculated data are assembled in Table 1.

### 2.4. The Aggregation Analysis of Enzymes with Ionic Liquids

The dynamic light scattering method was used to evaluate the effect of the ILs on the aggregation of the enzymes. Table 2 shows the hydrodynamic diameters of the enzymes and serum albumins upon the addition of the ILs. In all measurements, the concentration of the IL was 100 mM. The hydrodynamic diameters of lysozyme [33] and α-chymotrypsin [34] measured in this study are in good agreement with the data in the literature. For lysozyme and α-chymotrypsin, the addition of ILs does not lead to significant changes in their hydrodynamic diameter, indicating the absence of large aggregate formation.

### 2.5. The Molecular Docking Analysis of Enzymes with Ionic Liquids

**Figure 8:**
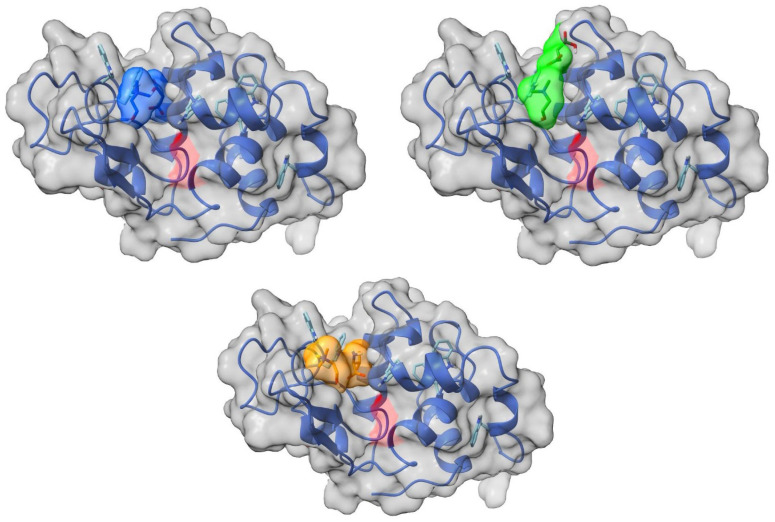
Localization of ionic liquids’ ions on the surface of the lysozyme. Top row: left: [Ch][Mal], right: [Ch][Suc], bottom row: [Ch][Val]. The surface of the active site of the enzyme is highlighted in red, the enzyme backbone is represented by the blue line, its surface is shown in grey, and the ions of the ionic liquid are shown in light blue ([Ch][Mal]), green ([Ch][Suc]), and yellow ([Ch][Val]).

The localizations of the ions of the ILs on the surface of a lysozyme globule, according to an analysis of their molecular docking, are presented in Figure 8. All ionic liquids are in the form of a tight ion pair and reside in the main binding grove of the lysozyme.

**Figure 9:**
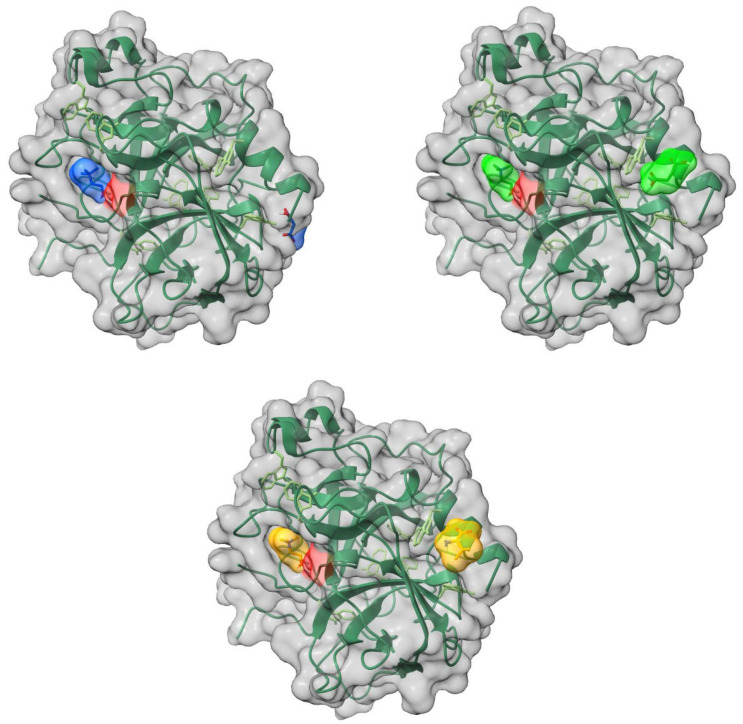
Localization of ionic liquids ions on the surface of the a-chymotrypsin. Top row: left: [Ch][Mal], right: [Ch][Suc], bottom row: [Ch][Val]. The surface of the active site of the enzyme is highlighted in red, the enzyme backbone is represented by the green line, its surface is shown in grey, and the ions of the ionic liquid are shown in light blue ([Ch][Mal]), green ([Ch][Suc]), and yellow ([Ch][Val]).

The cations and anions of the ILs are localized on separate sites on the surface of α-chymotrypsin (Figure 9). The cations of all of the studied ILs are located in the binding pocket of the enzyme. The anions are positioned on the opposite side of the globule.

The binding energies of ILs bound to the studied enzymes, estimated by molecular docking analysis, are presented in Table 3. There is virtually no difference in the binding energies of the studied ILs according to the docking data. On the other hand, α-chymotrypsin forms stronger complexes with the ILs than lysozyme.

The modeling results suggest that the ILs establish hydrogen bonds with the polar residues of Ser, Thr, and Asn in lysozyme and with the polar residues of Ser, Thr, Gln in α-chymotrypsin.

## 3. Discussion

Previously, we studied the effects of the same ionic liquids on albumins [26]. Interestingly, the studied ILs either did not affect the thermal stability of the albumins or increased it; however, an opposite effect was observed for both enzymes. Some ILs caused a blue shift in the albumin fluorescence, indicating an increase in the polarity of the chromophore microenvironment, which can be interpreted as tightening of the protein globule [35,36]. In contrast, no changes or a red shift in fluorescence were observed in the studied enzymes.

In the case of all studied proteins, [Ch][Suc] caused the smallest changes to the tertiary structure. At the same time, the effects of [Ch][Mal] and [Ch][Mal] were protein-specific.

As in the case of serum albumins, the binding constant of [Ch][Val] in binding to enzymes is significantly larger than those of [Ch][Suc] and [Ch][Mal]. However, the binding constants of the ILs bound to the enzymes are still relatively low, even though it is evident that the studied ILs can form hydrogen bonds through their carboxyl and/or amide groups. Moreover, the studied ILs have a pronounced negative effect on the thermal stability of the enzymes. It appears that the ILs may form hydrogen bonds with the water molecules surrounding the enzymes, thus destabilizing the enzyme structure. In addition, due to the decrease in intermolecular hydrogen bonding, the water becomes free to compete with the intramolecular interactions of biomolecules [37]. However, these effects should be largely the same for both the albumins and the studied enzymes. The observed differences likely indicate that the structure of the albumins is “tuned” to tolerate ligand addition. Interestingly, although albumins have lower thermal stability than lysozyme, ILs have a much more pronounced destabilizing effect on the latter.

No aggregate formation was observed upon the addition of ILs to the enzyme solution, whereas albumin aggregates in the presence of almost all ILs (with the exception of [Ch][Suc]/BSA). It was previously shown that the formation of BSA aggregates at 25 °C, upon IL addition, leads to an increase in the biomolecule’s thermostability. The differences in the proteins’ tendency to aggregate may also indicate that albumins and enzymes interact with small molecules in distinct ways. This can be rationalized as follows: The effect of the small molecules on the stability of the proteins can be viewed as the result of the two main factors: first, the effect of the medium, i.e., how the interactions of the small molecules with water alter the water’s properties as a solvent (e.g., by reducing the hydrophobic effect); second, the direct interaction of the small molecules with both native and denatured protein forms. Often, small molecules with hydrophobic moieties associate with unfolded protein molecules, thereby shifting the folding equilibrium towards unfolding. On the other hand, if the folded protein has a binding site for a specific small molecule (at least at moderate concentrations), the folded structure will be stabilized. Typically, enzymes have binding sites tuned to their specific substates, while albumins, as transport proteins, contain multiple binding sites that can accommodate a variety of molecules. Therefore, we hypothesize that small molecules are more likely to destabilize enzymes, unless they structurally resemble the enzyme’s specific substrate.

The fundamental differences in the behavior of enzymes and albumins in IL solutions can be attributed to their distinct structural and functional characteristics. Albumins, as transport proteins, possess a high degree of flexibility in binding various molecules, which increases the likelihood of aggregation. They contain multiple binding sites capable of accommodating a wide range of ligands, leading to potential conformational changes that promote aggregation, especially under excess ligand conditions. In contrast, enzymes have highly specific binding sites tailored to interact with particular substrates or cofactors, which reduces the chances of nonspecific interactions with solution components, including ILs, and consequently diminishes their tendency to aggregate even under destabilizing conditions.

## 4. Materials and Methods

### 4.1. Chemicals

Lysozyme and α-chymotrypsin (each with reported purities exceeding 99%) were obtained from Sigma-Aldrich (Burlington, VT, USA). Aqueous enzyme solutions containing ionic liquids (ILs) were prepared using deionized water (resistivity: 18 Ω·cm). The ILs had purities above 96%; for the details of their synthesis and characterization refer to [32].

### 4.2. Sample Preparation

The enzyme solutions in water/IL mixtures were prepared using the following protocols. For circular dichroism and dynamic light scattering measurements, solutions containing 5 mg/mL of enzyme were prepared by dissolving lyophilized enzymes in a corresponding amount of 10 mM of PBS buffer. The solutions were then equilibrated for 30 min at room temperature. Subsequently, the required amount of the IL was added to the enzyme solution to obtain the desired final concentration. For fluorescence measurements, the solutions were prepared similarly but with 1 mg/mL of enzyme.

### 4.3. Circular Dichroism Spectroscopy (CD)

To assess changes in the tertiary structure of the enzymes, CD spectra were recorded over the wavelength range of 250–310 nm using a J-1500 CD spectropolarimeter (Jasco, Tokyo, Japan). Enzyme solutions were prepared at 5 mg/mL in 10 mM of PBS. Temperature-dependent scans were performed in 5 °C increments at a heating rate of 5 °C/min, with an in-cell thermocouple used for temperature control.

### 4.4. Fluorescence Spectroscopy

Enzyme solutions (1 mg/mL) were prepared in 10 mM of PBS at varying IL concentrations. Fluorescence emission spectra were recorded on a Hitachi F-7000 spectrofluorimeter (Tokyo, Japan), using an excitation wavelength of 295 nm.

### 4.5. Hydrodynamic Size Measurements Using Dynamic Light Scattering

The hydrodynamic diameter of particles in the enzyme/IL solutions was measured using a Brookhaven Instruments 90 Plus Particle Size Analyzer (Nashua, NH, USA); the final diameter was determined as the average of three replicate measurements. A 5 mg/mL protein concentration was employed.

### 4.6. Molecular Docking

The interaction of the ionic liquids with α-chymotrypsin and lysozyme was investigated using Autodock Vina 1.2.3 and AutoDock Tools (ADT 1.5.6) [38]. Crystal structures of α-chymotrypsin (PDB: 1YPH) [link] and lysozyme (PDB: 6LYZ) [39] were retrieved from the RCSB Protein Data Bank [40]. Only one subunit of α-chymotrypsin was used (the other was removed). The grid box for α-chymotrypsin measured 48 × 56 × 50 Å, and for lysozyme it measured 44 × 44 × 48 Å, each with a spacing of 1.0 Å.

Multiple-ligand docking was carried out following the updated AutoDock Vina 1.2.3 guidelines [https://autodock-vina.readthedocs.io/en/latest/docking_multiple_ligands.html] (23 May 2019). In accordance with the protocol, each ionic liquid was represented as separate anion and cation species. The geometric optimization of each cation and anion was done using the PM3 method in MOPAC2016 [http://openmopac.net/Manual/index.html] (23 May 2019). The resulting OUT files were converted to PDBQT format using Open Babel 2.4.1 [41].

## 5. Conclusions

In the present study, the interaction of lysozyme and α-chymotrypsin with choline-based ionic liquids with biological anions was investigated. We conducted a comparative analysis of the behavior of these ionic liquids in both enzyme and albumin solutions. It was found that the thermostability of the enzymes, upon the addition of ILs, either remained the same as in their native form or decreased, whereas, in the case of serum albumin, an increase in thermostability was observed. Moreover, increasing the concentration of ILs in the enzyme solutions lead to a greater unfolding of the tertiary structure than in the previously studied serum albumin solutions. Based on fluorescence spectroscopy data, the binding constants of the ILs for the enzymes were calculated. It was demonstrated that the binding constants of the ILs with enzymes and those with albumins were similar. Furthermore, the correlation between the structure of the ionic liquids and the binding constants was the same for both the enzymes and albumins. However, it was found that the enzymes did not tend to form large aggregates in the IL solutions, whereas the albumins did form aggregates (with the exception of the [Ch][Suc]/BSA system).

The results underscore the need for a deeper understanding of the differences and similarities in the behavior of different ionic liquids in various environments. The distinct interaction patterns of ILs with albumins and proteins likely reflect the evolutionary “fine-tuning” of protein properties. Future research will hopefully help to identify structure–property relationships that can predict the stabilizing or destabilizing effects of ILs on biomolecules.

## Figures and Tables

**Table 1:** The binding parameters of biomolecules bound to ionic liquids.

Ionic Liquids	K_sv_/M^−1 a^	K_b_/M^−1 a^	*n*
Lysozyme			
[Ch][Mal]	4.88 ± 1.24	2.61 ± 1.32	0.71
[Ch][Suc]	22.62 ± 2.54	31.64 ± 6.01	1.15
[Ch][Val]	75.90 ± 8.96	73.84 ± 10.34	0.99
α-Chymotrypsin			
[Ch][Mal]	12.82 ± 2.87	6.6 ± 2.93	0.67
[Ch][Suc]	20.66 ± 4.21	30.95 ± 5.69	1.19
[Ch][Val]	128.72 ± 15.84	280.6 ± 30.05	1.40
HSA ^b^			
[Ch][Suc]	18.15 ± 0.92	26.36 ± 1.07	1.18
[Ch][Val]	64.12 ± 7.69	267.49 ± 1.17	1.54
BSA ^b^			
[Ch][Mal]	8.52 ± 1.58	2.57 ± 1.04	0.43
[Ch][Suc]	22.42 ± 2.04	110.46 ± 1.24	1.68
[Ch][Val]	113.16 ± 12.44	308.11 ± 1.31	1.38

^a^ Uncertainties in this table correspond to the expanded uncertainties (0.95 level of confidence k = 2). ^b^ Data were taken from [26].

**Table 2:** Hydrodynamic diameter ($d_{H}$) and polydispersity index (PDI) values of biomolecules with ionic liquids.

Ionic Liquids	*d_H_*/nm ^a^	PDI
Lysozyme		
Pure Lysozyme	3.3 ± 1.2	0.256
[Ch][Mal]	3.8 ± 1.4	0.290
[Ch][Suc]	7.4 ± 1.8	0.261
[Ch][Val]	5.3 ± 1.5	0.247
α-Chymotrypsin		
Pure a-Chymotrypsin	3.4 ± 1.1	0.259
[Ch][Mal]	5.4 ± 1.6	0.275
[Ch][Suc]	6.1 ± 1.6	0.284
[Ch][Val]	9.3 ± 2.5	0.273
BSA ^b^		
Pure BSA	7.71	0.302
[Ch][Mal]	47.23	0.315
[Ch][Suc]	6.55	0.254
[Ch][Val]	40.64	0.302
HSA ^b^		
Pure HSA	7.81	0.307
[Ch][Mal]	89.34	0.284
[Ch][Suc]	77.10	0.284
[Ch][Val]	97.67	0.298

^a^ Uncertainties in this table correspond to the expanded uncertainties (0.95 level of confidence k = 2). ^b^ Data were taken from [26].

**Table 3:** Thermodynamic stability of complexes of ILs with α-chymotrypsin and lysozyme obtained by molecular docking simulations.

Ionic Liquids	−G_bind_ (kcal/mol)	
	α-Chymotrypsin	Lysozyme (PDB Code: 6LYZ)
[Ch][Mal]	7.25	6.09
[Ch][Suc]	7.4	5.99
[Ch][Val]	7.24	6.15

## Data Availability

Data will be provided upon request.
